# Метастатическое поражение гипофиза

**DOI:** 10.14341/probl13395

**Published:** 2024-11-05

**Authors:** А. М. Лапшина, Е. А. Базарова, Е. Г. Пржиялковская, П. М. Хандаева, В. Н. Азизян, А. Ю. Григорьев, О. В. Иващенко, Н. В. Тарбаева, Ж. Е. Белая

**Affiliations:** Национальный медицинский исследовательский центр эндокринологии; Национальный медицинский исследовательский центр эндокринологии; Национальный медицинский исследовательский центр эндокринологии; Национальный медицинский исследовательский центр эндокринологии; Национальный медицинский исследовательский центр эндокринологии; Национальный медицинский исследовательский центр эндокринологии; Национальный медицинский исследовательский центр эндокринологии; Национальный медицинский исследовательский центр эндокринологии; Национальный медицинский исследовательский центр эндокринологии

**Keywords:** метастазы в гипофиз, масс-эффект, пангипопитуитаризм, гипопитуитаризм

## Abstract

Метастатическое поражение гипофиза является редким состоянием и диагностируется в 1,8–4% случаев. Авторы представляют трех пациентов с метастатическим поражением гипофиза, которые подверглись нейрохирургическому лечению в ГНЦ ФГБУ НМИЦ эндокринологии с последующим патоморфологическим подтверждением диагноза. Первичными опухолями были рак молочной железы, карциноид легкого, светлоклеточный рак почки. У двух пациентов были также обнаружены отдаленные метастазы, кроме гипофиза. Клиническая манифестация заключалась в появлении симптомов пангипопитуитаризма, хиазмального синдрома и масс-эффекта во всех случаях. Период наблюдения после хирургического лечения составил 0,25–2,5 года. У двух пациенток отмечено прогрессирование основного заболевания. Одной из них проводилось стереотаксическое радиохирургическое лечение и стереотаксическое облучение. У одного пациента состояние стабильно. Наблюдение и лечение таких пациентов является сложной задачей и требует повышенного внимания со стороны мультидисциплинарной команды специалистов.

## Abstract (en)

Metastatic lesion of pituitary is a rare condition and is diagnosed in 1.8–4% of cases. Monitoring and treatment of such patients is a complex task and requires increased attention from a multidisciplinary team of specialists. The authors represent three patients with metastatic pituitary lesion who underwent neurosurgical treatment at the National Research Center of the National Research Institute of Endocrinology with subsequent pathomorphological confirmation of the diagnosis. The primary tumors were breast cancer, lung carcinoid, and clear cell kidney cancer. Two patients had distant metastases other than the pituitary gland. The clinical manifestation consisted in the appearance of symptoms of panhypopituitarism, chiasmal syndrome and mass effect in all cases. The follow-up period after surgical treatment was 0.25–2.5 years. Progression of the underlying disease was noted in two patients. One of them carried out stereotactic radiosurgical treatment and stereotactic oriented irradiation. One patient has a stable condition.

## ВВЕДЕНИЕ

Гипофиз является достаточно редкой мишенью для распространения метастазов из различных первичных злокачественных опухолей. Процесс метастазирования и непосредственно сама патология недостаточно изучены.

Известно, что среди всех интракраниальных метастазов вторичные очаги в гипофизе составляют 3%, в операционном материале частота диагностики достигает 1,8%, по данным аутопсий, несколько больше — до 4% у пациентов с известным онкологическим заболеванием [1, 2].

В систематическом обзоре Sam Ng et al. [2] показано, что наиболее распространенные злокачественные опухоли, которые метастазируют в гипофиз, были рак легких (31%) и рак молочной железы (26%). Третье место занимает рак почки (8,1%).

Распределение по полу в достаточной мере не изучено. Однако известно, что рак легких является наиболее частой первичной опухолью у мужчин, метастазирующей в гипофиз, а рак молочной железы — у женщин. Более редкими первичными новообразованиями являются опухоли печени, желудочно-кишечного тракта, меланомы, лимфомы и др. Возраст пациентов, страдающих от данной патологии, составляет от 50 до 60 лет.

Выживаемость достаточно низкая, в среднем около 12–14 мес. На период выживаемости влияют такие факторы, как локализация первичной опухоли и распространенность процесса, проведение радиотерапии или химиотерапии. Известно, что период выживаемости при наличии метастазов в гипофизе рака молочной железы или рака почки гораздо выше (до 22–30 месяцев) по сравнению с опухолями легкого.

Мы представляем серию пациентов с метастатическим поражением гипофиза, которые подверглись нейрохирургическому лечению в ГНЦ ФГБУ НМИЦ эндокринологии с последующим патоморфологическим подтверждением диагноза.

## КЛИНИЧЕСКИЙ СЛУЧАЙ 1

Пациентка С., 34 года, поступила с жалобами на снижение зрения с височной стороны, общую слабость, утомляемость, периодически головные боли, отсутствие менструаций. Летом 2018 г. пациентка самостоятельно обнаружила уплотнение в левой молочной железе, по поводу которого в течение года наблюдалась по месту жительства. По данным трепанбиопсии опухоли от июля 2019 г., выявлены структуры инвазивной протоковой карциномы II степени злокачественности. Проводилось иммуногистохимическое исследование, выявлена экспрессия HER2 (3+++), индекс Ki67 составил 30%. Проведено всего 8 курсов химиотерапии. При динамическом наблюдении, по данным КТ грудной клетки и органов брюшной полости (декабрь 2019 г.), выявлено метастатическое поражение печени и тела поясничного позвонка L1. Далее, в марте 2020 г., выполнена радикальная мастэктомия по Мадену слева. По данным патоморфологического исследования, выявлен лечебный патоморфоз 1–2 степени злокачественности инвазивной карциномы G II с метастазом в 1 из 10 лимфоузлов.

С октября 2020 г. стала отмечать ухудшение общего самочувствия в виде сухости, жжения и покраснения в глазах, спустя время отметила двоение в глазах, появились головные боли преимущественно в теменной области. Выполнено МРТ головного мозга в ноябре 2020 г., в хиазмально-селлярной области выявлено кистозно-солидное объемное образование размерами 22х23х13 мм с компрессией хиазмы, умеренной деформацией зрительных трактов; деформирует дно третьего желудочка, требуется проведение дифференциальной диагностики с краниофарингиомой, однако ранее по МРТ головного мозга от августа 2019 г. образование не визуализировалось (рис. 1.1). При госпитализации в ГНЦ ФГБУ «НМИЦ эндокринологии» Минздрава в декабре 2020 г. у пациентки подтвержден гипопитуитаризм: вторичная надпочечниковая недостаточность (кортизол утром — 96,16 нмоль/л (171–536), АКТГ утром — 13,86 пг/мл (7,2–63,3)), вторичный гипотиреоз (ТТГ — 1,59 мМЕ/л (0,25–3,5), свТ4 — менее 5,15 пмоль/л (9–19)), гипогонадотропный гипогонадизм (ФСГ — 2,21 Ед/л (1,9–11,7), ЛГ — 0,22 Ед/л (2,6–12,1), эстрадиол — 58,07 пмоль/л (97–592)). Данных за гормональную активность образования (пролактин — 263,5 мЕд/л (64–395), ИФР-1 — 199,45 нг/мл (78–311) и нарушение функции нейрогипофиза не получено. При осмотре офтальмологом выявлена частичная атрофия зрительных нервов, при периметрии сужение полей зрения не зафиксировано. Проведено МСКТ органов грудной клетки, очаговых и инфильтративных изменений в паренхиме легких не определяется. Ранее, в ноябре 2020 г., проводилась сцинтиграфия костей всего тела, визуализировалось избыточное накопление индикатора в области правой половины тела L1, пациентка на момент поступления получала золедроновую кислоту по 4 мг в/в 1 раз в 1,5 месяца. В отделении инициирована терапия гидрокортизоном, левотироксином, в связи с раком молочной железы, от назначения менопаузальной гормональной терапии принято решение воздержаться. В связи с развитием масс-эффекта опухоли (головные боли, снижение зрения) пациентке выполнено трансназальное транссфеноидальное хирургическое вмешательство в декабре 2020 г. В операционном материале среди ткани аденогипофиза имелись участки опухоли в виде гнезд и кластеров (рис. 1.2), при иммуногистохимическом исследовании клетки опухоли негативны к транскрипционным факторам гипофиза, гормонам гипофиза, эстрогеновым и прогестероновым рецепторам, позитивные к цитокератину 7 (рис. 1.3, 1.4). Т.е. гистологическая картина и иммунофенотип соответствовали метастазу неспецифицированного рака молочной железы.

У данной пациентки период между постановкой диагноза первичной опухоли и выявлением метастаза в гипофиз составил около 24 месяцев. На момент обнаружения новообразования в гипофизе у пациентки подтвержден распространенный метастатический процесс с выявлением вторичных очагов в левой гемисфере мозжечка, печени, позвонке L1. Далее, в 2021 г., дважды проведено стереотаксическое радиохирургическое лечение с прогрессированием заболевания. В 2022 г. выполнено дистанционное стереотаксическое ориентированное облучение на аппарате «гамма нож». На фоне приема 90 мг преднизолона отмечено развитие экзогенного гиперкортицизма и остеопении в поясничных позвонках и в проксимальном отделе бедренной кости после облучения. Длительность наблюдения с момента установления метастаза в гипофиз составляет 31 месяц.

**Figure:**
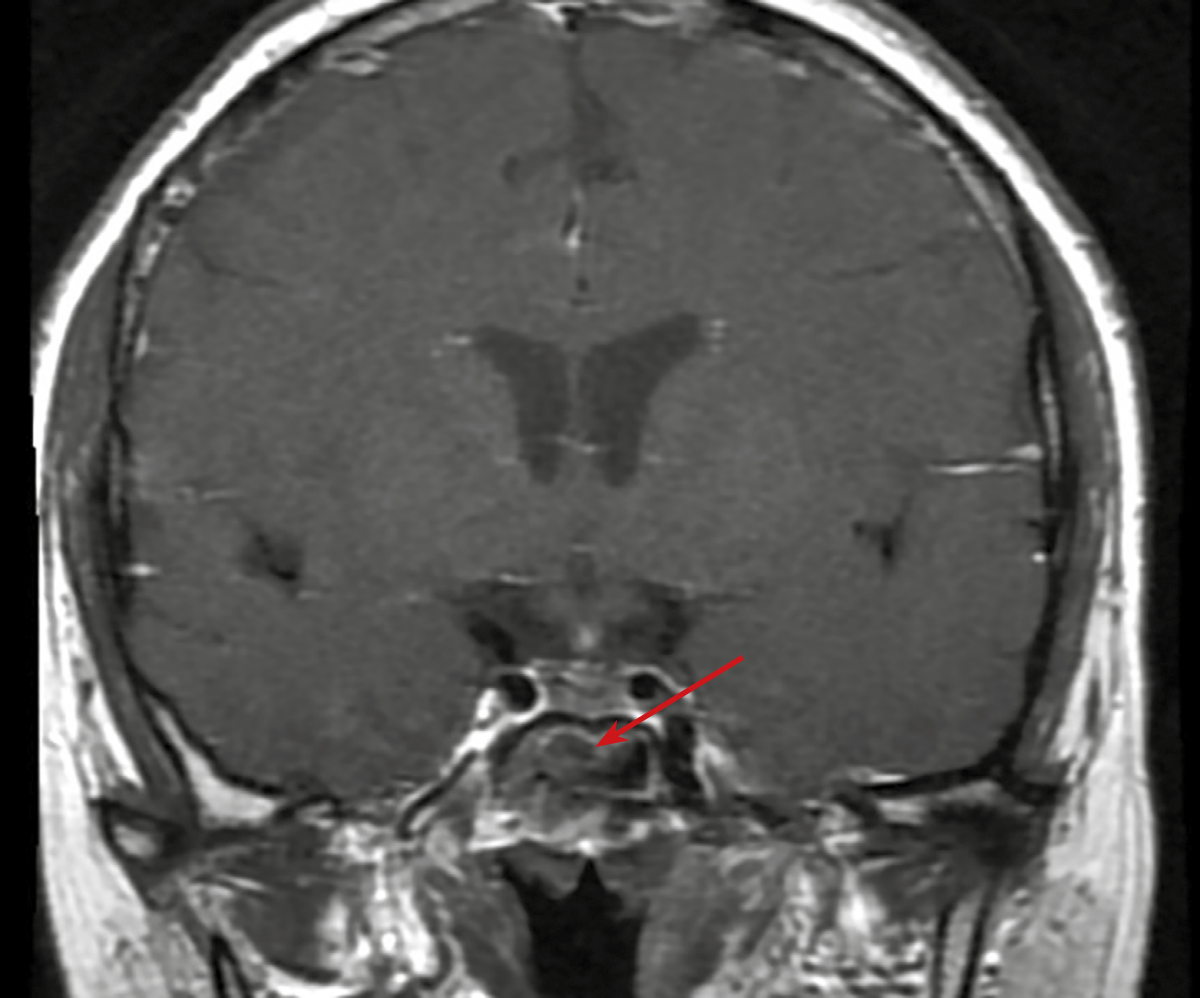
Рисунок 1.1. Кистозно-солидное объемное образование гипофиза с супра- и латероселлярным ростом влево, с компрессией хиазмы, умеренной деформацией зрительных трактов и третьего желудочка.

**Figure:**
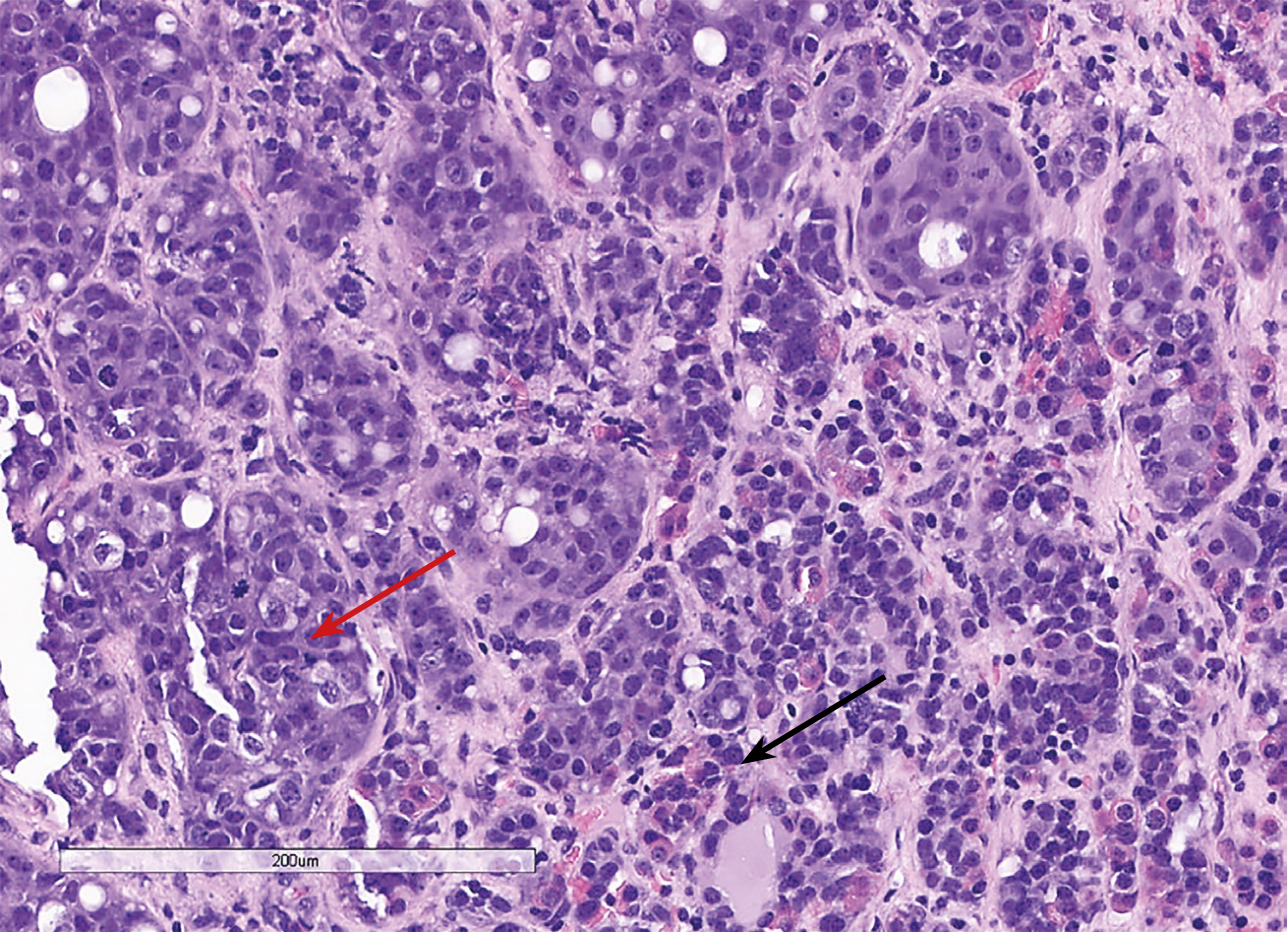
Рисунок 1.2. Окраска гематоксилином и эозином. Среди элементов опухоли (красная стрелка) определяются фокусы аденогипофиза (черная стрелка).

**Figure:**
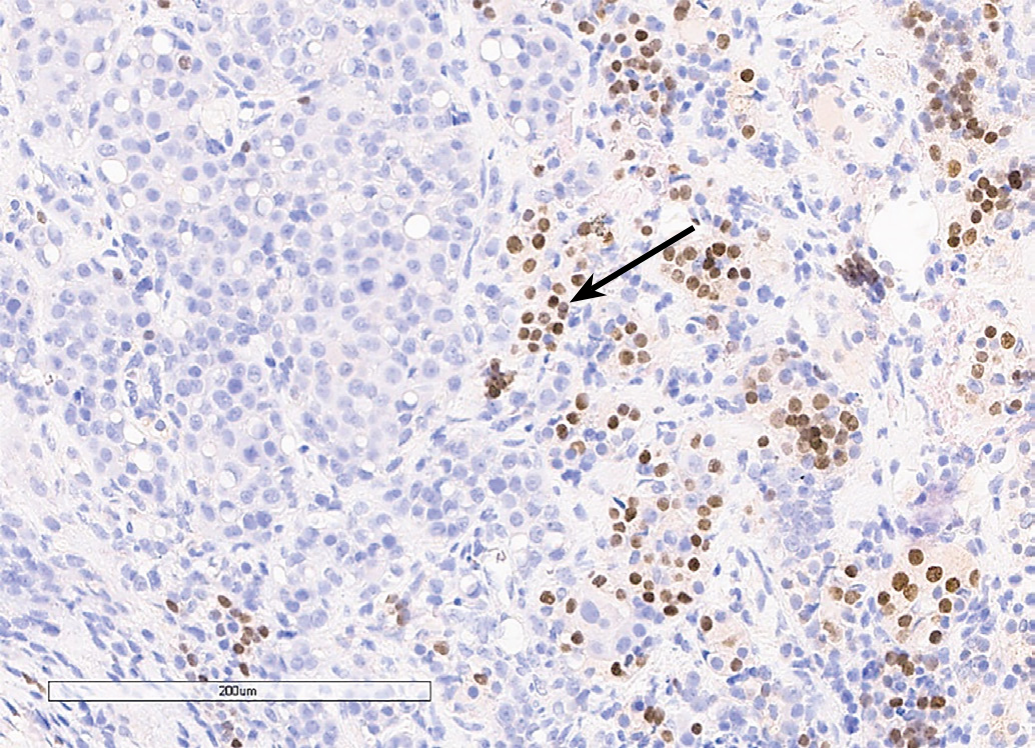
Рисунок 1.3. Иммуногистохимическое исследование: отсутствие окрашивания с антителами к транскрипционному фактору PIT1 в клетках опухоли при окрашивании клеток аденогипофиза (черная стрелка).

**Figure:**
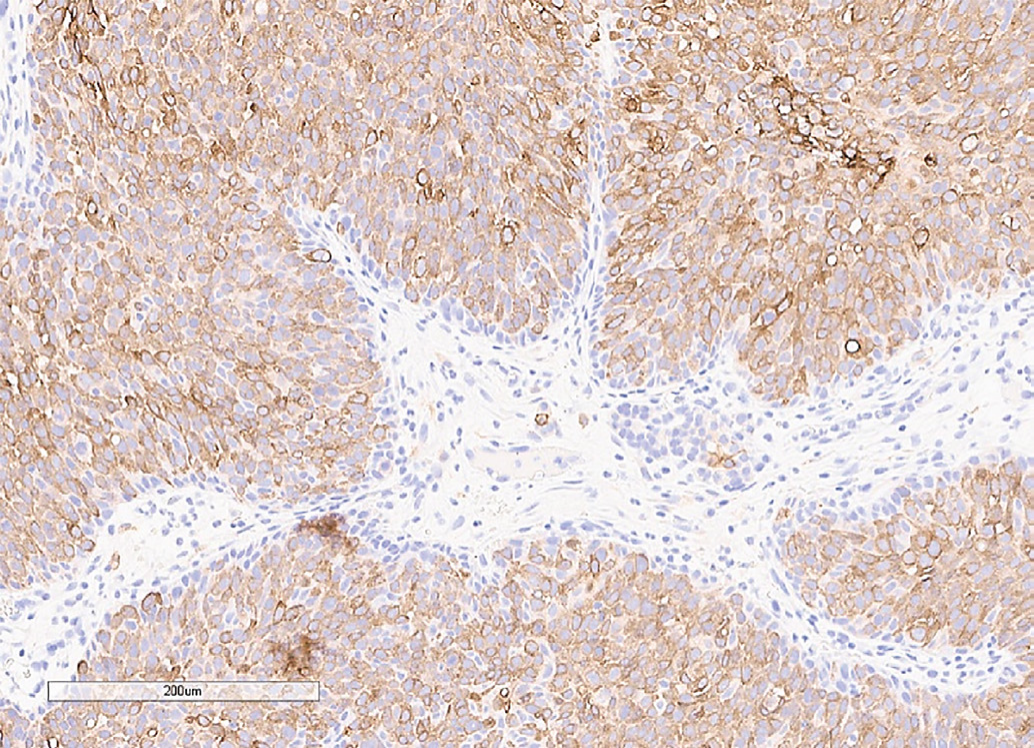
Рисунок 1.4. Иммуногистохимическое исследование: окрашивание с антителами к СК 7 в клетках опухоли.

## КЛИНИЧЕСКИЙ СЛУЧАЙ 2

Пациентка К., 73 лет, поступила с жалобами на головную боль, ухудшение зрения, общую слабость.

Из анамнеза известно, что в 2012 г. перенесла комплексное лечение по поводу типичного карциноида правого легкого Т1N1M0 IIA, выполнена средняя лобэктомия справа с медиастинальной лимфаденэктомией, дистанционная лучевая терапия. По данным иммуногистохимического исследования, маркер Ki67 составил 2%.

В 2021 г. появились головные боли. В октябре 2021 г., по данным МРТ, выявлена макроаденома гипофиза, проводился осмотр офтальмолога, зарегистрировано снижение остроты зрения.

С марта-апреля 2022 г. отметила усиление головных болей, снижение зрения, выраженную общую слабость. В марте 2022 г., по данным МРТ, подтверждено объемное образование хиазмально-селлярной области размером 31х20х33 мм с супра- и параселлярным распространением. Впервые, по данным гормонального обследования, при госпитализации в ГНЦ ФГБУ «НМИЦ эндокринологии» Минздрава в марте 2022 г. выявлена умеренная гиперпролактинемия (пролактин — 1356,2 мМЕ/л при норме до 557, повышенного уровня макропролактина не выявлено), назначена терапия каберголином. Также лабораторно отмечены низкие значения гонадотропинов (ЛГ — 0,13 Ед/л, ФСГ — 3,08 Ед/л) у пациентки в менопаузе, что говорит о наличии вторичного гипогонадизма.

В мае 2022 г. впервые госпитализирована в ГНЦ ФГБУ «НМИЦ эндокринологии» Минздрава России. При гормональном обследовании убедительных данных за гормональную активность опухоли гипофиза не получено: исключен гиперкортицизм (кортизол в суточной моче — 68 нмоль/л (при норме до 379), акромегалия (ИФР-1 — 166,4 нг/мл (норма — до 238 нг/мл)), пролактинома (пролактин на фоне 5-дневного приема 0,25 мг каберголина в сутки — 175 мЕД/л (норма до 340). У пациентки установлен диагноз вторичной надпочечниковой недостаточности: уровень кортизола крови утром — 170 нмоль/л. Инициирована терапия глюкокортикоидами. Данных за вторичный гипотиреоз (ТТГ — 4,29 мМЕ/л (0,25 — 3,5), свТ4 — 14,69 пмоль/л (9–19)) и нарушение функции нейрогипофиза не получено.

Проведен осмотр офтальмолога: по данным периметрии, выявлено сужение полей зрения концентрическое на оба глаза на 10–20 градусов. Оптимальным методом лечения выбрана трансназальная транссфеноидальная аденомэктомия.

Из значимой сопутствующей патологии: в 2017 г. установлен первичный гиперпаратиреоз, проведено хирургическое лечение, при госпитализации в июне 2022 г. подтверждена ремиссия, пациентка получает антирезорбтивную терапию по поводу остеопороза.

Принимая во внимание карциноид легкого в анамнезе, проведено КТ органов грудной клетки с контрастированием: образования в 9-м ребре слева и 7-м ребре справа максимальным размером до 3,3 см, для дифференциальной диагностики между бурыми опухолями, метастазами карциноида, рекомендовано проведение соматостатин-рецепторной сцинтиграфии.

Учитывая сочетание нескольких нейроэндокринных опухолей, рекомендовано генетическое исследование для исключения синдрома множественной эндокринной неоплазии 1 типа, однако установлен диагноз фенотипически. В рамках поиска других опухолей проводилась КТ органов брюшной полости: признаков нейроэндокринных опухолей поджелудочной железы, образований надпочечников не обнаружено. По результатам лабораторного обследования, данных за наличие активной нейроэндокринной опухоли нет: гастрин — 69,4 пг/мл (13,0 — 115,0), хромогранин А — 0,90 нмоль/л (<2,00), инсулин — 13,68 мкЕ/мл (2,6 — 24,9).

Пациентке выполнено эндоскопическое трансназальное удаление опухоли гипофиза в июне 2022 г.

По данным патоморфологического исследования, выявлены фрагменты опухоли из железистоподобных структур, клетки которых обладали признаками нейроэндокринной дифференцировки (рис. 2.1). По данным иммуногистохимического исследования, в клетках опухоли обнаружена экспрессия TTF-1, хромогранина А, низкомолекулярного цитокератина (клон САМ 5.2), что свидетельствовало о наличии метастаза нейроэндокринной опухоли легкого. Экспрессия гормонов гипофиза (АКТГ, СТГ, ПРЛ, ТТГ, ЛГ, ФСГ) отсутствует. Индекс метки Ki-67 составил 12,2% (рис 2.2, 2.3).

У данной пациентки период между постановкой диагноза первичной опухоли карциноида легкого и выявления метастаза в гипофиз составил около 108 месяцев. На ПЭТ КТ с 18Ф-ФДГ (декабрь 2022 г.) обнаружен очаг в S6 правого легкого, единичные очаги во внутригрудных лимфоузлах, очаги в костях скелета, в том числе в ребрах. На момент написания статьи длительность наблюдения пациентки составляет 12 месяцев.

**Figure:**
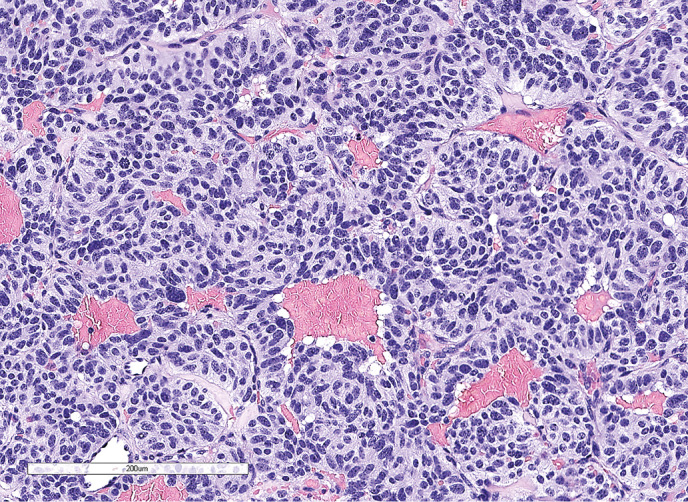
Рисунок 2.1. Окраска гематоксилином и эозином. Железисто-подобные структуры и клетки опухоли с признаками нейроэндокринной дифференцировки.

**Figure:**
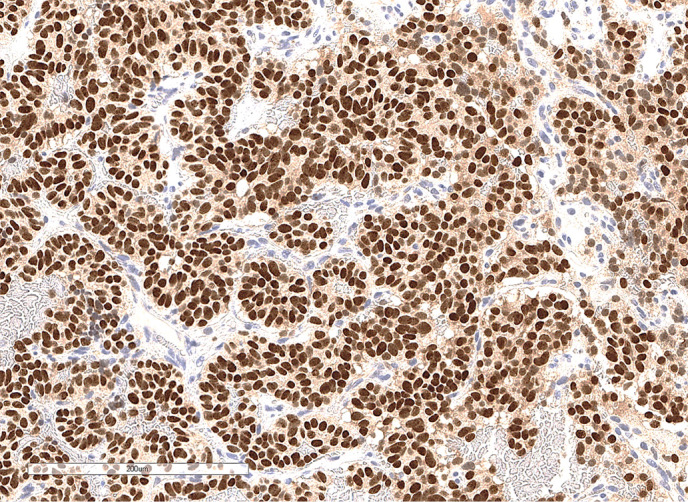
Рисунок 2.2. Иммуногистохимическое исследование: окрашивание ядер опухолевых клеток с антителами к TTF-1.

**Figure:**
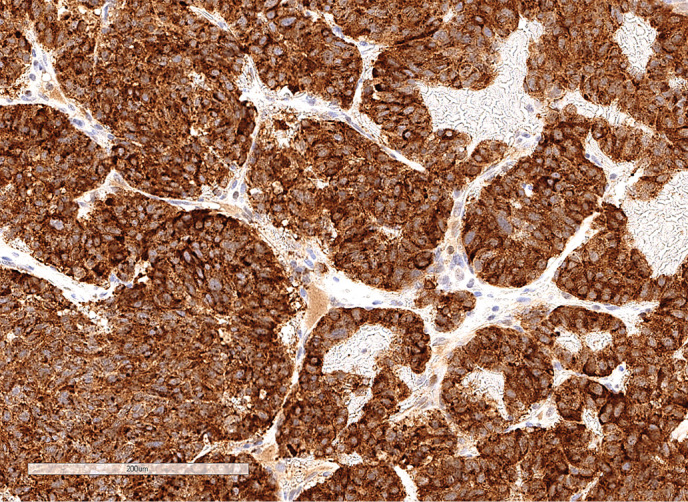
Рисунок 2.3. Иммуногистохимическое исследование: окрашивание цитоплазмы опухолевых клеток с антителами к хромогранину А.

## КЛИНИЧЕСКИЙ СЛУЧАЙ 3

Пациент Г., мужчина 73 лет, отметил появление выраженных зрительных нарушений (снижение остроты зрения на левый глаза, двоение) с последующим ухудшением. Данная симптоматика возникла через 6 лет после радикальной левосторонней нефрэктомии в 2017 г., выполненной по поводу светлоклеточного почечноклеточного рака (рТ2N0M0). По данным компьютерной томографии через 4 года (2021 г.), выявлена опухоль в правой почке 14х15х14 мм солидной структуры. В январе 2022 г. в связи с потерей сознания госпитализирован в городскую больницу по месту жительства, где диагностирована вторичная надпочечниковая недостаточность. Назначена заместительная терапия препаратами преднизолона. Впервые в феврале 2022 г. выполнена КТ головного мозга, обнаружены признаки объемного образования хиазмально-селлярной области, пролабирующего через верхнюю апертуру турецкого седла с ровными контурами, размером 10х12х14 мм, параселлярные структуры без особенностей. МРТ не проводилась в связи с клаустрофобией у пациента. Во время госпитализации в ГНЦ ФГБУ НМИЦ эндокринологии в марте 2022 г. подтверждена вторичная надпочечниковая недостаточность (АКТГ <5,00 пг/мл (0–46), кортизол <1,00 мкг/дл (5–25), а также установлен гипогонадотропный гипогонадизм (тестостерон — 3,22 нг/дл (167–654), ФСГ — 0,32 мМЕ/мл (0,95—11,95). При обследовании была исключена гормональная активность опухоли при наличии умеренного повышения уровня общего пролактина до 1243 мЕд/л, что было интерпретировано как масс-эффект опухоли. По результатам МСКТ забрюшинного пространства с контрастным усилением, данных за рецидив заболевания не получено, сохраняется образование правой почки, без отрицательной динамики. При повторной госпитализации через 1 год (март 2023 г.) отмечалась отрицательная динамика в виде прогрессирования зрительных нарушений и роста образования хиазмально-селлярной области до 18х27х15 мм с признаками сдавления хиазмы и пролабирования в полость 3 желудочка на 7,2 мм, компримируя его передние отделы (рис. 3.1).

Пациент осмотрен офтальмологом, выполнена компьютерная периметрия, выявлено сужение периферической границы поля зрения концентрическое на 10 градусов справа, с височной стороны на 15–20 градусов слева. При обследовании данных за наличие метастазов почечноклеточного рака другой локализации не получено.

Пациенту выполнено трансназальное трансфеноидальное удаление опухоли в марте 2023 г. Операционный материал был представлен фрагментами ткани опухоли из клеток с оптически светлой цитоплазмой, расположенные в тонкой сосудистой сети (рис. 3.2), позитивные к CD10 при иммуногистохимическом исследовании (рис. 3.3). Ткань нейро- и аденогипофиза не получена. Диагностирован метастаз светлоклеточного почечноклеточного рака.

У данного пациента период между постановкой диагноза первичной опухоли и выявления метастаза в гипофиз составил 53 месяца. Данные о других метастазах у пациента отсутствуют. Период наблюдения пациента составляет 18 месяцев, после проведенного хирургического лечения — 4 месяца.

Основные клинические характеристики пациентов суммированы в таблице 1.

**Figure:**
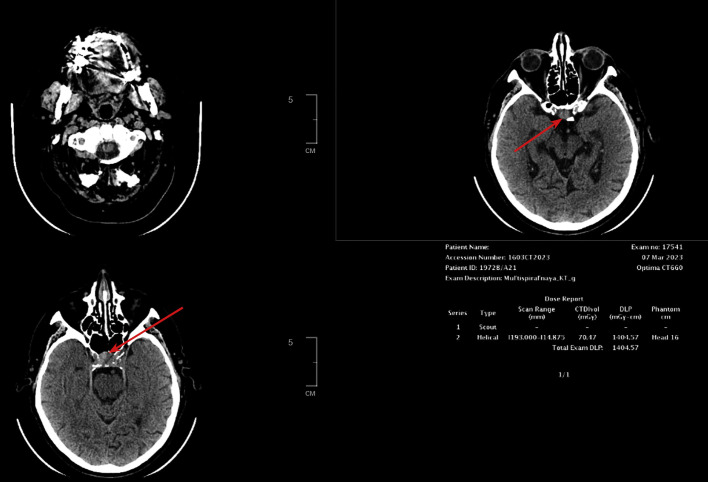
Рисунок 3.1. КТ головного мозга.

**Figure:**
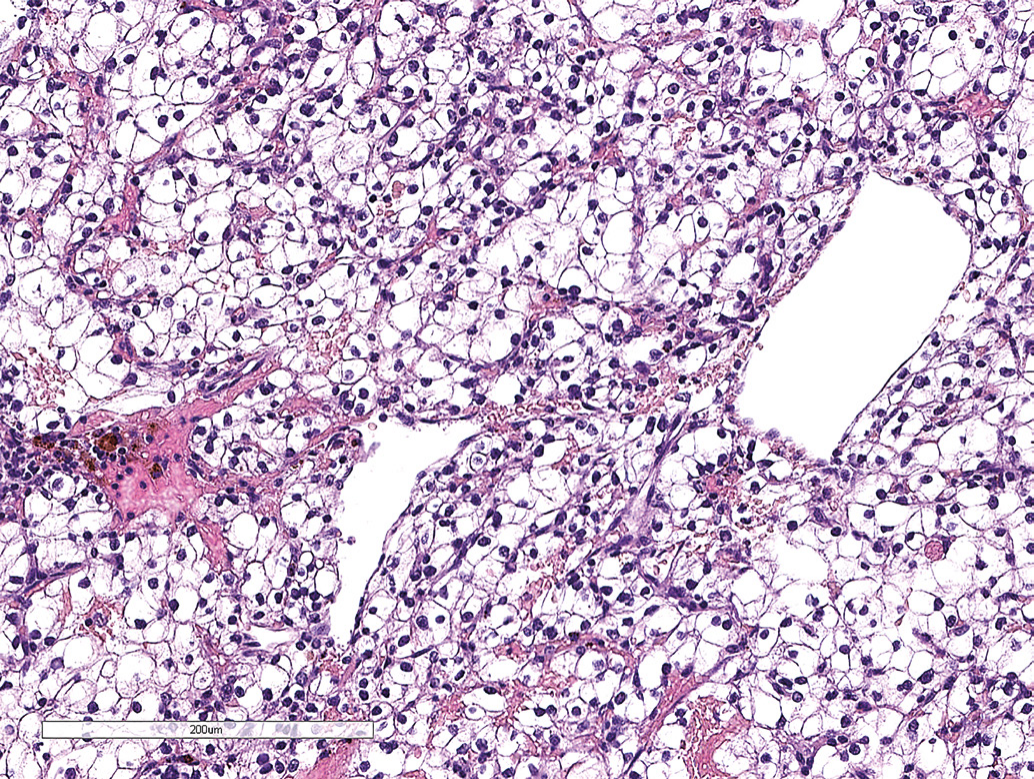
Рисунок 3.2. Окраска гематоксилином и эозином. Метастаз светлоклеточного почечноклеточного рака.

**Figure:**
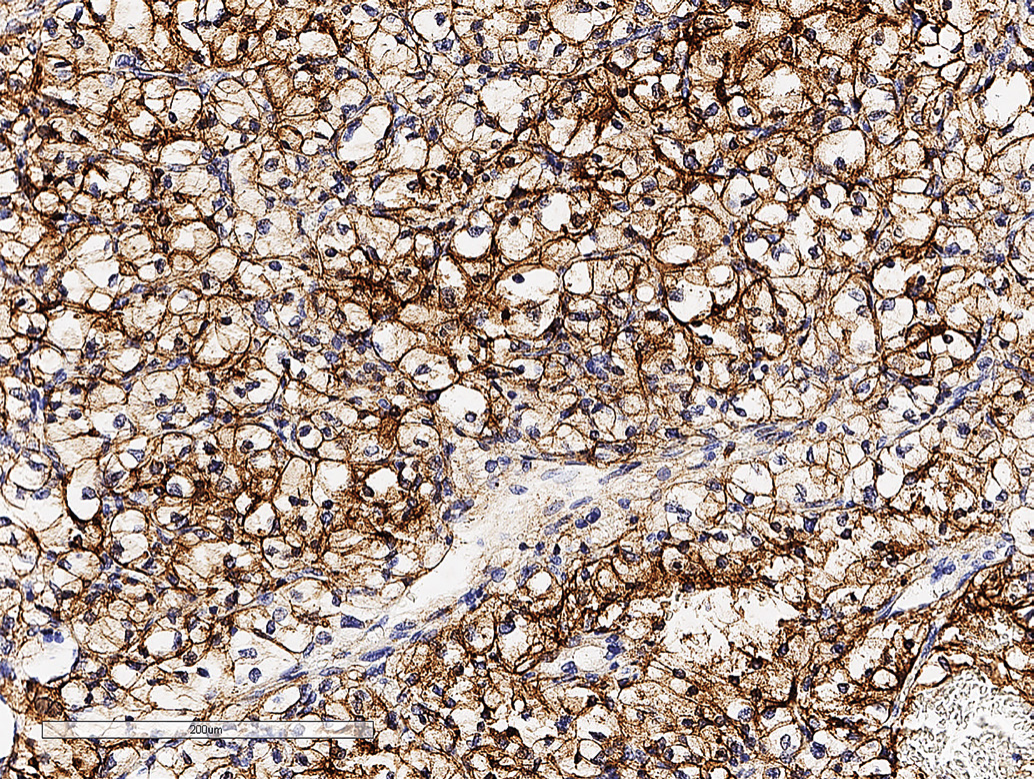
Рисунок 3.3. Иммуногистохимическое исследование: окрашивание клеток опухоли с антителами к CD10.

**Table:** Таблица 1. Клиническая характеристика пациентов Сокращения: РМЖ — рак молочной железы, СПР — светлоклеточный почечноклеточный рак, НХ — нейрохирургия.

№	пол	Возраст манифестации (годы)	Первичная опухоль	Наличие других метастазов	Максимальный размер метастаза в гипофизе (мм)	Клиническая картина	Период наблюдения после НХ (годы)
1	ж	32	РМЖ	да	23	Пангипопитуита-ризм, зрительные нарушения	2,5
2	ж	61	Карциноид легкого	да	33	Пангипопитуита-ризм, зрительные нарушения	1,0
3	м	67	СПР	нет	27	Пангипопитуита-ризм, зрительные нарушения	0,25

## ОБСУЖДЕНИЕ

Представлены клинические случаи метастатического поражения гипофиза при различных первичных злокачественных опухолях (рак молочной железы, карциноид легкого, светлоклеточный рак почки), подтвержденные гистологически. Период между диагностикой первичной опухоли и метастазов в гипофиз составил от 24 до 216 месяцев (1–9 лет). Во втором и третьем клинических случаях пациенты находились, в целом, в длительной ремиссии по поводу основного заболевания на момент диагностики метастатического поражения гипофиза. Однако пациентка с раком молочной железы проходила множественные курсы химиотерапии по поводу распространенного метастатического процесса. На дооперационном этапе у одного пациента диагностирован центральный несахарный диабет, все пациенты отмечали снижение зрения и головные боли, был подтвержден гипопитуитаризм. Полученные данные в целом совпадают с данными литературы. Так, клиническая манифестация метастатического поражения гипофиза наблюдается примерно у 18% пациентов. Наиболее часто исследователи отмечают развитие несахарного диабета, хиазмального синдрома, нарушение функции передней доли гипофиза в виде частичного выпадения секреции гормонов либо пангипопитуитаризма, а также неспецифического признака — головной боли [3]. В серии наших пациентов практически у всех наблюдалось выпадение функции передней доли гипофиза, головная боль и хиазмальный синдром. Размеры новообразования хиазмально-селлярной области, выявленные в нашем исследовании, в целом совпадают с другими работами.

По данным МРТ, средний диаметр вторичных очагов гипофиза составил 21,2±10,3 мм (n=318), у 22,3% зафиксировано распространение опухоли в кавернозный синус [2][4][5].

В отличие от первичных опухолей гипофиза, которые развиваются в передней доле, метастазы чаще встречаются в задней доле и воронке. По данным различных авторов, в 57,0–84,6% случаев поражается только задняя доля или в комбинации с передней, в то время как передняя доля — в 13,0–15,4%. Вероятно, частота поражения задней доли обусловлена гематогенной диссеминацией метастазов и особенностями кровоснабжения нейро- и аденогипофиза. Кровоснабжение передней доли гипофиза в основном происходит за счет гипофизарной портальной системы с наличием 2 капиллярных сплетений, что, предположительно, является своеобразным препятствием для распространения опухолевых клеток, а задней доли — непосредственно из нижней гипофизарной артерии [3]. Вместе с тем у пациентов, описанных в нашей серии, преимущественно выпадала функция передней доли гипофиза.

Для проведения декомпрессии хиазмально-селлярной области всем пациентам выполнено трансназальное транссфеноидальное нейрохирургическое вмешательство. Основным методом верификации метастатического поражения гипофиза является патоморфологическое исследование. Образование хиазмально-селлярной области в сочетании с быстро прогрессирующим нарушением зрения, головными болями, центральным несахарным диабетом и гипопитуитаризмом у пациентов старше 50 лет или у пациентов с онкологическим анамнезом подразумевает исключение метастатического поражения гипофиза, даже несмотря на длительную ремиссию ранее известного онкологического заболевания [5][6]. По данным Sam Ng et al., в изучаемой когорте у 58,6% пациентов уже известна локализация и гистогенез первичной опухоли. Метастатический статус подтвержден только в 21,9% случаев [3].

Среди методов лечения возможно выбрать нейрохирургическое удаление метастатического очага гипофиза, радиотерапию, химиотерапию или таргетную терапию. В целом выживаемость не отличается среди пациентов, которые подверглись нейрохирургическому лечению, и теми, кого лечили нехирургическими методами. В пользу выбора хирургического метода служит быстрое устранение компрессии хиазмы и получение ткани опухоли для патоморфологического исследования. В случае обширного распространения метастаза в кавернозные синусы и другие структуры тотальная резекция не приводит к увеличению периода выживаемости. Потенциально предпочтительными являются радиотерапия, что связано с тенденцией к росту выживаемости, переносится пациентами легче и дает меньше осложнений по сравнению с хирургическим методом или химиотерапией. Химиотерапия или таргетная терапия могут рассматриваться в качестве дополнительных методов лечения, которые предполагают повышение периода выживаемости [7][8].

Прогноз у таких пациентов в целом неблагоприятный и в первую очередь зависит от типа первичной опухоли и распространенности процесса. Молодой возраст, небольшой объем поражения гипофиза и короткий период между диагностикой первичного злокачественного новообразования и метастазами в гипофиз может ассоциироваться с более длительным периодом выживаемости [9-11].

## ЗАКЛЮЧЕНИЕ

Таким образом, несмотря на редкость метастатического поражения гипофиза, у врачей должна сохраняться настороженность, чтобы не пропустить потенциально жизнеугрожающее состояние гипопитуитаризма и вторичной надпочечниковой недостаточности, компенсация которых позволяет продлить жизнь и улучшить качество жизни пациентов.

## ДОПОЛНИТЕЛЬНАЯ ИНФОРМАЦИЯ

Источники финансирования. Работа выполнена при поддержке гранта РНФ: 19-15-00398-П.

Конфликт интересов. Авторы декларируют отсутствие явных и потенциальных конфликтов интересов, связанных с содержанием настоящей статьи.

Участие авторов. Все авторы одобрили финальную версию статьи перед публикацией, выразили согласие нести ответственность за все аспекты работы, подразумевающую надлежащее изучение и решение вопросов, связанных с точностью или добросовестностью любой части работы.

Авторы настоящей статьи получили письменное согласие от пациентов на публикацию медицинских данных, упоминаемых в статье.
